# Laboratory Assessment of Unfractionated Heparin (UFH) with Activated Clotting Time (ACT) and Anti-Xa Activity during Peripheral Arterial Angiographic Procedure

**DOI:** 10.3390/diagnostics13081489

**Published:** 2023-04-20

**Authors:** Tuukka Helin, Tomi Tirri, Heidi Korkala, Kimmo Lappalainen, Lotta Joutsi-Korhonen

**Affiliations:** 1 Department of Clinical Chemistry, HUS Diagnostic Center, Helsinki University Hospital, P.O. Box 720, 00029 Helsinki, Finland; 2 Department of Clinical Chemistry, University of Helsinki, 00014 Helsinki, Finland; 3 Department of Radiology, HUS Diagnostic Center, Helsinki University Hospital, P.O. Box 320, 00029 Helsinki, Finland

**Keywords:** activated clotting time, anti-Xa, endovascular, interventional radiology, unfractionated heparin

## Abstract

Activated clotting time (ACT) is used in cardiac surgery for monitoring unfractionated heparin (UFH). In endovascular radiology, ACT use is less established. We aimed to test the validity of ACT in UFH monitoring in endovascular radiology. We recruited 15 patients undergoing endovascular radiologic procedure. ACT was measured with ICT Hemochron^®^ device as point-of-care (1) before standard UFH bolus, (2) immediately after the bolus, and in some cases (3) 1 h into the procedure or a combination thereof (altogether 32 measurements). A total of two different cuvettes, ACT-LR and ACT+ were tested. A reference method of chromogenic anti-Xa was used. Blood count, APTT, thrombin time and antithrombin activity were also measured. UFH levels (anti-Xa) varied between 0.3–2.1 IU/mL (median 0.8) and correlated with ACT-LR moderately (R^2^ = 0.73). The corresponding ACT-LR values were 146–337 s (median 214). ACT-LR and ACT+ measurements correlated only modestly with one another at this lower UFH level, with ACT-LR being more sensitive. Thrombin time and APTT were unmeasurably high after the UFH dose, rendering them of limited use in this indication. We adopted an ACT target of >200–250 s in endovascular radiology based on this study. While ACT correlation with anti-Xa is suboptimal, the readily available point-of-care nature increases its suitability.

## 1. Introduction

Peripheral arterial angiographic procedures are often required in atherosclerosis obliterans when critical limb ischemia develops in order to regain function and ultimately prevent limb loss [1]. Intravenous unfractionated heparin (UFH) is given as an antithrombotic during angiographic procedures as prophylaxis for arterial thrombosis. Standard bolus dosing is most often used with 5000 IU dose recommended for complex vascular procedures [2]. However, there are individual differences in the effect, predisposing to thrombotic and bleeding complications. Heparin confers its anticoagulant activity by inhibiting thrombin and factor Xa with antithrombin as a mediator. Heparin resistance can in some patients massively impact the required heparin dose, and functional testing for heparin effect is required to identify this phenomenon. Thus, individual monitoring and dose tailoring are likely to improve outcomes and patient safety.

Traditionally, UFH monitoring is performed with coagulation testing in plasma. That is, using activated partial thromboplastin time (APTT) and anti-Xa. However, the narrow measurement range and long turnaround times of these tests are limiting their use perioperatively, where rapid decisions based on the coagulation testing are required. While the above-mentioned APTT and anti-Xa are well established, a methodology with point-of-care, rapid results would facilitate heparin dosing during procedures. Activated clotting time (ACT) is a whole blood measurement performed bedside, with wide measurement range, enabling UFH monitoring at various therapeutic dosages. ACT has been used as point-of-care test since the 1960s in cardiac surgery to monitor UFH anticoagulation during cardiopulmonary bypass [3]. ACT is less sensitive to heparin than APTT, enabling monitoring of very high concentrations used during cardiac surgery. Yet, the whole blood matrix in the ACT differs from the plasma commonly used in coagulation assays, with potential interferences of clotting factor deficiencies and anticoagulants other than heparins. In addition, in contrast to APTT, there are no well-established target values for ACT, but instead, local target values based on device and experience are used for UFH titration. Universal target values may be difficult to achieve, due to varying devices in use, as well as the clinical situations of the patients being treated. Most commonly, values above 480 s are recommended during cardiopulmonary bypass. These values were obtained during the early use of the assay, when it was discovered, that in the setting of cardiopulmonary-bypass, ACT levels of at least 400 s were required to achieve coagulation-free bypass [4,5]. The large unphysiological surfaces during cardiopulmonary bypass are not present during endovascular peripheral arterial procedures, thus making the treatment range for ACT significantly lower. Yet, in these indications, ACT use or target ranges are not well established. There are, however, guidelines recommending administration of UFH and measurement of ACT during endovascular peripheral arterial procedures [1], with a common target value being usually approximately 200–250 s [1,6,7,8,9]. However, in different hospitals, localized protocols are often in use, as evidenced by the varying ACT targets even in cardiopulmonary bypass, with the longest experience with the assay [10]. While different ACT coagulometers generally provide similar results, different ACT devices have varying sensitivities to UFH as well as coagulation factor levels and other patient-related factors, supporting the use of localized ACT target ranges [11]. There is no reference material or gold standard for ACT testing.

We aimed to assess the feasibility of ACT assessment on the effects of UFH in patients undergoing angiographic procedure at our hospital. Both ACT and comparative coagulation tests anti-Xa and APTT from plasma were assessed to gain comparative data in different patients. Further, we examined the effects of preoperative hemoglobin and platelet count on the ACT.

## 2. Materials and Methods

### 2.1. Patients

At the time of this study, at our department of Radiology, patients undergoing angiographic procedures were treated with unfractionated heparin (UFH, Heparin Leo^®^), with standard heparin bolus of 5000 IU regardless of the weight of the patient, without laboratory monitoring. In this study, ACT and other coagulation tests were performed with 15 patients and altogether 32 blood samples were taken. Participants were recruited from patients treated by the same interventional radiologist, to limit the interindividual variation in the procedures. Patient characteristics, indications and UFH doses used are shown in Table 1. Of the patients, two received low-molecular-weight heparin (LMWH) before the procedure, but this had no impact on the baseline ACT values (137 s and 149 s, ACT-LR median 142 s in all patients for the baseline) and anti-Xa was 0 IU/mL at this point. The median age of the 15 patients was 62 years (range 32–93) and the median weight was 83 kg (range 55–132 kg). Only two (13%) were women. Samples were taken either (1) prior to the procedure, (2) immediately after UFH bolus of 5000 IU, (3) during the procedure, prior to the next UFH bolus, (4) after subsequent UFH bolus of 2500–4000 IU. Measurements were not time-matched, clinical situation determined the timing of the ACT. More than one bolus of UFH was given when needed, if the procedure was prolonged.

Venous blood samples were collected from catheter into a syringe and, immediately 15 uL of the whole, non-anticoagulated blood was applied on the ACT Hemochron cuvette and 2.7 mL into a vacuum test tube with 3.2% sodium citrate (BD Vacutainer^®^) for other coagulation tests. The test tube was mixed 4–5 times thoroughly, centrifuged with local standard procedures (2500 g, 10 min) and coagulation testing was performed within 2 h.

The study received institutional approval (HUS/628/2019). Patients gave informed consent for participation in the study. Our study was non-invasive, and no extra samples were required, as patient heparin monitoring was performed with anti-Xa assay. The ACT tests measured had no impact on patient treatment, as during the study, anti-Xa was used for dose estimation.

### 2.2. Methods

ITC Hemochron Signature Elite^®^ (application version v2.2, bios version v2.2) device and ACT-LR and ACT+ cuvettes were used for measuring ACT (Instrumentation Laboratory). A 15 uL volume of untreated, non-anticoagulated whole blood is needed for one measurement of ACT.

In the ACT-LR cuvette the activator is celite, and in the ACT+ a mixture of kaolin, silica and phospholipids. Celite is known to be more sensitive to heparin and ACT-LR cuvette is recommended, when the expected ACT value would be below 400 s. When expected ACT values are above 400 s (such as during cardiopulmonary bypass) ACT+ cuvettes are required. In this study, both cuvettes were tested for this indication to appreciate which better correlates with the anti-Xa assay. No patients received UFH preoperatively, with 2/15 (13%) receiving LMWH prior to the procedure.

The manufacturer recommends using ACT-LR at UFH levels up to 2.5 IU/mL and ACT+ at 1.0–6.0 IU/mL. Since the UFH level was not definitively known prior to the study, both cuvettes were tested in parallel. The Hemochron^®^ device uses optical LED-sensors to assess blood clotting—when the blood flow slows in the channel below a specified cut-off, a clot is formed and clotting time in seconds is shown.

The measuring range for coagulation times in the Hemochron^®^ device ACT measurement ranges from 0–1005 s. Coefficients of variation (CV) for the assay were calculated using normal, healthy volunteers: CVs for ACT, as well as for prothrombin (PT) and APTT cuvettes were performed using 5 repeat samples from 5 donors: CV 11.1%, 6.9% and 14.4%, respectively. Liquid DirectCheck Controls provided by the manufacturer level 1 (normal) and level 2 (abnormal) were used for quality control.

As reference, chromogenic anti-FXa activity (Hemosil Liquid Anti-Xa^®^), with same calibration suitable for both LMWH and UFH measurement, was used to measure UFH level, the analyzer used was ACL TOP 750^®^ (Instrumentation Laboratory). The measuring range was 0.04-4.0 IU/mL, local CV of the assay was 6.4%. Antithrombin activity (Siemens Berichrom Antithrombin III^®^), thrombin time (Siemens BC Thrombin Reagent^®^), the APTT (Siemens Actin FSL^®^) and the PT (Axis-Shield Nycotest PT^®^) were assessed with BCS XP^®^ coagulation analyzer (Siemens Healthineers, Erlangen, Germany). To obtain blood counts, EDTA anticoagulated samples were analysed by Sysmex XE-2100 hematology analysers by routine procedures at the HUSLAB Laboratory Services.

### 2.3. Statistical Methods

Pearson correlations and Bland–Altmann plots were assessed for comparison. Repeatability of assays was estimated using simple coefficient of variation (CV) calculations. The statistical analyses were performed with IBM SPSS Statistics^®^ (version 25).

## 3. Results

**Figure 1:**
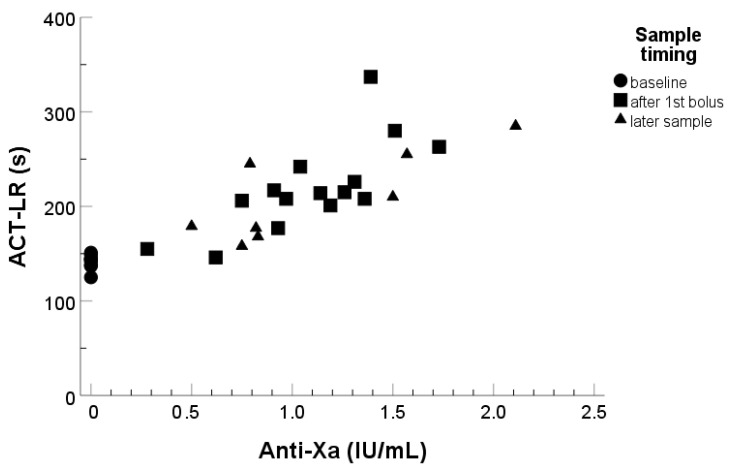
Correlation between activated clotting time (ACT, Hemochron^®^) and UFH level measured with anti-FXa (IL^®^), *n* = 32 different measurement points in 15 patients after IV heparin treatment undergoing peripheral arterial angiographic procedure. Correlation with low-range ACT-LR measurement, R^2^ = 0.73. Correlation with high-range ACT+-measurement, R^2^ = 0.70.

The ACT-LR baseline as well as values after UFH infusion are shown in Figure 1. At baseline, all the patients had their ACT-LR below 200 s. After the first UFH bolus (in most cases 5000 IU), 12/15 (80%) of patients had an ACT-LR of over 200 s and 3/15 (20%) had ACT-LR of over 250 s. The lowest anti-Xa value corresponding to ACT-LR over 200 s was 0.8 IU/mL. Of the patients, a high proportion (18/25) (72%) had an ACT value under the common target value of 250 s at first or subsequent measurements after the initial UFH bolus.

**Figure 2:**
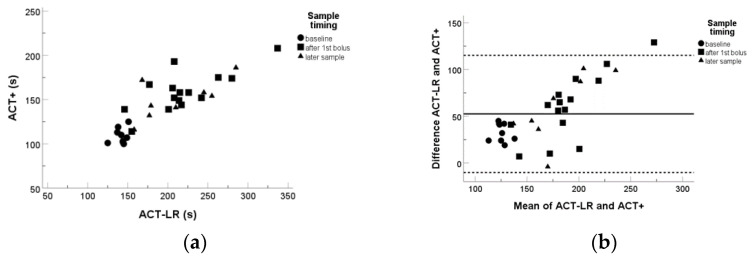
ACT-LR and ACT+ (Hemochron ^®^) correlated with one another fairly well R^2^ = 0.70 (**a**). ACT-LR was consistently more prolonged than ACT+ when measured in parallel. Differences increased after heparin bolus, Bland–Altmann plot with mean for difference (solid line) and ± 1,96 SD (dashed lines) are shown (**b**).

**Figure 3:**
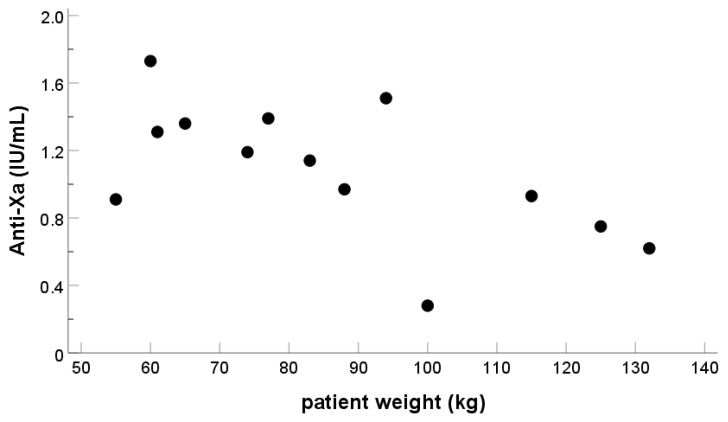
UFH level measured with chromogenic anti-FXa (IU/mL) after 5000 IU bolus and weight of the patient (kg), UFH level after 5000 IU UFH bolus. The correlation between patient weight and anti-Xa measurement was poor (A), R^2^ = 0.38. The correlation with ACT-LR measurement was even poorer R^2^ = 0.22.

Both ACT-LR and ACT+ correlated reasonably well with anti-Xa measurements as well as with one another, while ACT-LR expectedly, being recommended to use with lower UFH concentrations, gave higher prolongation in the patient samples with moderate UFH concentrations (Figure 1 and Figure 2). Patient weight had poor correlation with both anti-Xa and ACT-LR measurements (Figure 3), suggesting that other factors also influence the coagulation response.

After the first heparin bolus, only 1/13 patients had TT below the upper measurement limit of 140 s, while the same patient had his APTT within the local reference interval (23–33 s). TT and APTT were not measured at baseline. APTT was of little benefit after the 1st heparin bolus, as 8/14 patients had APTT above the measurement range of 180 s. Anti-Xa exceeded 0.2 IU/mL in all the samples after heparin administration. Antithrombin activity was measured at baseline and was always normal preoperatively, enabling heparin anticoagulant activity.

Preoperative hemoglobin (Hgb) values were within the reference intervals in 9/15 (60%) patients, with one patient (6%) having Hgb in reference range measured only after the procedure. A total of four (27%) patients had Hgb below the reference interval preoperatively, all were men. Hgb values did not correlate with ACT-LR results (R^2^ = 0.03). The preoperative platelet count was within the reference interval in all 15 patients and did not correlate with ACT-LR results (R^2^ = 0.02). A total of five (33%) patients were followed for hemoglobin postoperatively, studied within one month of the procedure. Of these, one woman with AV malformation procedure and one man with DVT thrombectomy had significant change in Hgb. The Hgb result was significantly lower (> 2.0 g/dL) than preoperatively and slightly below the reference intervals. Yet, the drop in Hgb in these patients could not be directly attributed to the procedure as complicating factors (upper limb AV malformation and DVT treatment) were present. As the Hgb did not correlate with ACT-LR, heparinization alone does not account for the drop in Hgb. However, the majority of patients, 10/15 (67%), had no Hgb measurements within one month postoperatively. Indeed, at least 13/15 (87%) had no significant bleeding postoperatively.

Due to the whole blood matrix of the ACT assay, platelet counts, and thrombocytopenia might in principle affect the results of the ACT. The platelet counts of the patients were measured preoperatively, and none of the patients had thrombocytopenia.

## 4. Discussion

The main findings of our study are that ACT can be used to monitor heparin response in patients during interventional radiology procedures. While anti-Xa assay is most accurate, ACT assay correlated moderately well with the anti-Xa assay, making this rapidly available assay suitable for heparin monitoring during the procedure. Modern assays are also easy to use. Since no centrifugation is required, assays can be performed from whole blood by clinicians and nurses participating in the procedure, without requiring laboratory technicians or other dedicated staff. Further, the device manufacturers generally recommend easily managed control schemes with liquid quality control samples.

ACT is commonly used to monitor UFH in high thrombosis and bleeding risk procedures, such as cardiopulmonary bypass and vascular surgery. However, ACT, anti-Xa or indeed, any coagulation measurement is used much less in interventional radiology procedures. In a UK survey, only 4% of interventional radiologists measured UFH response with a clotting method (interpreted as ACT in the survey report), while in a survey in the Netherlands, 15% measured UFH with clotting assay (most likely ACT) [2,12]. Further, even when ACT is used, it is not commonly compared to traditional APTT or anti-Xa testing. In the US between 2001–2007, ACT was not used in the majority of centers, with limited data on its use thereafter [6]. In 2007, the co-operation between medical and surgical vascular, cardiovascular, vascular radiology and cardiology societies produced a consensus document on peripheral arterial disease, where ACT monitoring for UFH effect was recommended [1]. Yet, the utility of ACT measurement is still debated, with viewpoints for and against published recently [13,14]. Universal guidelines on ACT target levels, however, remain a challenge, as different devices are known to differ in their sensitivities to heparin as well as to patient-related confounders (e.g., Hgb, fibrinogen, FVIII levels, other anticoagulants) [15]. Local testing and verifications of ACT devices are required to further understanding of the use of this assay in the local patient population. Indeed, even in cardiac surgery, with decades of experience on ACT use, there is significant variation on the therapeutic ranges used. While ranges above 400 s are generally used, local variations are commonly implemented.

Outside surgical procedures and intensive care, where rapid turn-around time is of the essence, the APTT, thrombin time and anti-Xa assays are commonly used to monitor UFH effect [16,17,18]. The correlation of ACT to these tests has been previously studied with limited patient groups (peripheral arterial endovascular procedures, extracorporeal membrane oxygenation support patients) [19,20]. In clinical practice, it is uncommon to directly compare ACT with APTT or anti-Xa assays. This in in part due to the most common indication for ACT measurement being cardiac surgery. In these clinical situations, ACT may indeed be the only feasible measurement for heparin effect. This is due to the fact that with the highest concentrations of UFH in cardiac surgery settings, the APTT may become unmeasurable (that is, prolonged outside measurement range). However, in peripheral vascular procedures, with lower UFH dosages, it would be feasible to measure the UFH effect with anti-Xa or APTT, with the main factor supporting the ACT use being rapid turnaround time.

APTT and anti-Xa measurements are well established in the monitoring of UFH therapy. While anti-Xa has been selected as the preferred assay during endovascular procedures in some centers, the limiting factor of anti-Xa used is the slower turnaround time as compared to ACT [19]. The ACT can be performed bedside at point of care within a few minutes. This is of benefit, especially if the procedure is prolonged and additional coagulation measurements are required. The APTT has disadvantages of higher interpatient variability not related to the heparin dose as opposed to anti-Xa [21]. Here, the APTT was often prolonged above the measurement limit in our patients after UFH bolus of 5000 IU, rendering it of limited use in this indication. UFH anti-Xa response varied between 0.3–2.1 IU/mL after UFH bolus, with ACT-LR correlating moderately with anti-Xa assay. There was a wide variation in responses to the standard dose, most likely due to gender, weight and varying clinical situations of the patients. Previous LMWH doses in two patients did not influence the ACT-LR baselines, and anti-Xa was not detectable, where LMWH was used before the intervention. The lowest anti-Xa value corresponding to ACT-LR over 200 s was 0.8 IU/mL, strengthening the notion that ACT-LR of over 200 s might be a useful cutoff for UFH response in radiological interventions. The full range of ACT values of 125–337 s with highest anti-Xa of 1.7 IU/mL confirms the validity of using ACT-LR cuvettes during these procedures. ACT+ cuvette provided no additional information with poorer sensitivity at these UFH levels, and its use is discouraged in this indication. The limitation of ACT assays to consider in clinical interpretation, however, is that ACT behaves similarly to APTT in that its prolongation is not specific to heparin effects. Indeed, coagulation factor deficiency, and anticoagulants other than heparin may prolong the ACT. Further, due to the whole blood matrix of the assay, hemoglobin levels or platelet counts may also influence the measurement, while those have no effect on the APTT or anti-Xa. Nevertheless, with the recognition of these caveats, the ACT provides a rapid method to assess UFH response in these patients.

There are several potential limitations in this study. Firstly, ACT is not standardized and results are given as prolongation of coagulation time, whereas anti-Xa is standardized, giving results as UFH units. Secondly, anemia might affect the results, due to the whole blood sample matrix effect. Anemia might also affect hemostasis, independent of the coagulation activity, as red blood cells participate in primary hemostasis as well. In our study group, 4/15 (27%) patients had low hemoglobin but their ACT-LR at baseline did not differ when compared with the whole patient group. This corresponds well to the previously reported incidence of anemia. The incidence of preoperative anemia is quite high, in all procedures, even nearly 40%, and in one study with lower limb critical ischemia patients close to 20% [22,23]. At follow-up, most patients were not further tested for hemoglobin, suggesting that no major bleeding occurred. Yet, no firm conclusions can be made, due to the relatively limited patient population in this study. Further research with larger patient cohorts is required to further elucidate the effects of anemia to ACT in radiological interventions. Platelet counts were normal in all of the patients included in this study, so effects of platelet count on ACT could not be elucidated. In a previous in vitro study, decreasing hematocrit had only a mild effect on the ACT, while platelet count had no effect. Platelet activation with ADP and collagen had no significant effect on the ACT, while platelet fragmentation had a mild effect [24]. When using the ACT in patients with anemia, thrombocytopenia, or otherwise impaired primary hemostasis, it should be considered, however, that limited data exist on the influence of these preanalytic factors on the assay. Management of anemia and thrombocytopenia prior to procedure, where feasible, will enable most accurate heparin effect monitoring using the assay. An uncommon preanalytic factor is FXII deficiency, which causes marked prolongation of both APTT and ACT. The condition does not cause bleeding tendency, but excludes the use of APTT and ACT for heparin therapeutic monitoring in these patients [25]. Preoperative APTT testing will reveal this condition. The heterogeneous composition of this patient group limits the conclusions, as we included a patient with renal impairment and vascular malformation patients. The individual patient groups were too small to draw any conclusions on them. In patients undergoing percutaneous peripheral vascular intervention, preoperative anemia was common (42%) and associated with greater likelihood of adverse outcomes [26]. Thirdly, the relatively small number of recruited patients precludes inclusion of clinical outcome to the study. However, after the study was completed, ACT remains in use in our hospital during angioplasty, supporting the clinical work.

## 5. Conclusions

We have shown that ACT had moderate correlation with anti-Xa assay while monitoring UFH in a radiological endovascular procedure. While anti-Xa assay would be the most accurate for UFH dose response assessment, based on our study as well as previous research on the area, the practicality and accessibility with short turnaround time of ACT assay support its use during radiological interventions [19,20,27]. The rapid turnaround time enables multiple measurements during the procedure, when needed, and facilitates individual dosing. In some hospitals, the choice may be between ACT monitoring, and no monitoring at all, as the long turnaround time of APTT or anti-Xa precludes the use of these assays. Indeed, as coagulation monitoring is carried out during radiological vascular interventions, adoption of ACT should be encouraged with locally implemented target values for adequate UFH dose [2,12]. Yet, as previously discussed, many patient-specific factors, including coagulation factor deficiencies and impaired primary hemostasis may affect the ACT measurement, and preoperative laboratory screening is prudent to account for these factors and safe use of the ACT for heparin monitoring. Based on this study, at our hospital, we have decided to use the ACT target of 200–250 s, tailored to the procedure and patient bleeding risk, for UFH monitoring during angioplasty procedures.

## Figures and Tables

**Table 1:** Data of 15 patients undergoing endovascular procedure and receiving unfractionated heparin (UFH) as thromboprophylaxis.

Patient characteristics (*n* = 15)			
	Age, years median (range)	62 (32–93)	
	Gender (men/women)	13/2	
	Weight, kg, median (range)	83 (55–132)	
	Receiving aspirin/clopidogrel	9/4	
		N	%
Indication for the endovascular procedure			
	Lower limb artery stenosis (left/right side)	11 (8/3)	73
	Vascular malformation, AV upper limb or pulmonary	2	13
	Iliacal thrombectomy, deep vein thrombosis (right side)	1	7
	Dialysis fistula malfunction	1	7
UFH doses received			
	Only single dose 5000 IU	10	67
	Single dose 5000 IU and subsequent dose 2500–3000 IU	2	13
	Single dose 5000 IU, subsequent, third dose 2500–4000 IU	2	13
	Single dose 2500 IU (dialysis patient)	1	7
LMWH medication before procedure			
	Yes	2	13
	No	13	87

AV, arteriovenous. IU, international unit. LMWH, low molecular weight heparin. UFH, unfractionated heparin

## Data Availability

The data presented in this study are available on request from the corresponding author.
